# Autologous whole-tumor antigen vaccination in combination with adoptive T cell therapy for patients with recurrent ovarian cancer

**DOI:** 10.1186/2051-1426-1-S1-P220

**Published:** 2013-11-07

**Authors:** Lana E Kandalaft, Cheryl Chiang, Emese Zsiros, Janos Tanyi, Daniel Powell, Rosemarie Mick, George Coukos

**Affiliations:** 1 Ovarian Cancer Research Center, University of Pennsylvania, Philadelphia, PA, USA; 2 Department of Biostatistics and Epidemiology, University of Pennsylvania, Philadelphia, PA, USA

## 

Novel therapeutic strategies are warranted in recurrent ovarian cancer. We report two independent consecutive studies of combinatorial immunotherapy comprising dendritic cell (DC)-based autologous whole tumor antigen vaccination in combination with antiangiogenesis therapy. 31 patients with recurrent progressive stage III and IV ovarian cancer with available tumor lysate from secondary debulking surgery enrolled in two different studies. First 6 underwent priming with intravenous bevacizumab and oral metronomic cyclophosphamide followed by vaccination with an autologous DC preparation pulsed with freeze-thaw autologous tumor lysate while the other 25 underwent vaccination with an enhanced vaccine where autologous DCs were loaded with HOCl-oxidized autologous tumor lysate administered intranodally every 2 weeks in combination with intravenous bevacizumab. Both studies were followed by lymphodepletion and transfer of autologous vaccine-primed, ex vivo CD3/CD28-costimulated peripheral blood T-cells, in combination with antiangiogenesis therapy and vaccination. Feasibility, safety, and biological and clinical efficacy were evaluated. Eleven subjects have completed vaccination and T cell transfer to date, while twenty-three additional subjects completed vaccination only. Vaccination was well tolerated and elicited tumor-specific T cell responses against various ovarian tumor antigen in both studies and a clinical benefit of 65% correlated with the immune response with some experiencing prolonged progression free survival. Preliminary results demonstrate that patients’ DCs loaded with HOCl-oxidized lysate elicited strong tumor-specific IFN-γ secretions when incubated with autologous T cells post vaccination. These DC's also produced high levels of Th1-priming cytokines and chemokines, including IL-12. Following lymphodepletion, adoptive transfer of vaccine-primed T-cells was well tolerated and resulted in durable reduction of T-regulatory cells and restoration of vaccine-induced antitumor immunity in patients who experienced clinical benefit. eight out of 11 patients exhibited clinical benefit with one patient achieving a complete response at end of study. Stable disease was observed in 7 subjects to date. Data on additional patients will be presented at the meeting. Our results suggest the use of combinatorial cellular immunotherapy comprising DC vaccination with whole tumor antigen and adoptive lymphocyte transfer using tumor antigen-specific T cells for the treatment of patients with recurrent ovarian cancer is promising yet warrants further investigation.

